# Small Bowel Obstruction as a Complication of Acute Pancreatitis

**DOI:** 10.7759/cureus.74800

**Published:** 2024-11-29

**Authors:** Ahmed Mahmood, Siti Abdul Rahman, Shay Min Chua, Wael Abdelgawwad

**Affiliations:** 1 Accident and Emergency, Pilgrim Hospital, United Lincolnshire Hospitals Trust, Boston, GBR

**Keywords:** acute pancreatitis, acute small bowel obstruction, complicated acute pancreatitis, ischemic bowel, pancreatitis complications, splenic infarcts

## Abstract

Epigastric pain and vomiting are common presentations associated with various causes of acute abdomen. Acute abdomen encompasses a range of different pathologies, with epigastric pain narrowing the differential diagnosis to conditions such as pancreatitis, bowel obstruction, acute cholecystitis, gastritis, acute coronary syndrome (ACS), and peptic ulcer disease, such as gastric ulcers and duodenal ulcers with/without perforation.

This is a case of a male patient in his 80s who came to the emergency department with symptoms of generalized abdominal pain, vomiting, and constipation. Initial investigations and imaging supported a diagnosis of acute pancreatitis complicated by bowel obstruction. The patient was subsequently referred to the surgical team for further management.

## Introduction

According to the National Institute for Health and Care Excellence (NICE), acute pancreatitis is defined as an acute inflammatory process of the pancreas with varying involvement of local tissues or more remote organ systems. Acute pancreatitis is most commonly caused by gallstones or alcohol misuse, which accounts for around 75% of cases [1,2]. Other risk factors include post-endoscopic procedures, trauma, surgery, hyperglyceridaemia, hypercalcemia, drugs such as diuretics/statin/fenofibrate/angiotensin-converting enzyme inhibitors, chronic pancreatitis, anatomical disorders, autoimmune conditions, pancreatic malignancy [1,2]. In the UK, most hospitals serving a population of 300,000-400,000 people will admit approximately 100 people with acute pancreatitis per year [1].

Multiple complications from acute pancreatitis are infrequent but add complexity to both diagnosis and management, as demonstrated in this case. Small bowel obstruction secondary to acute pancreatitis is rare, often resulting from retroperitoneal inflammation and the close proximity of the small bowel to the anterior surface of the pancreas. An enzyme-rich extravasation product, released in response to the inflammatory process, travels to the colon and small bowels, mechanically and functionally obstructing bowel pathways [3].

## Case presentation

A gentleman in his 80s presented with a one-day history of abdominal pain, vomiting, reduced appetite, and a three-day history of constipation. On physical examination, he exhibited generalized abdominal tenderness with guarding and absent bowel sounds, suggesting an acute surgical abdomen with intestinal obstruction as our primary differential diagnosis.

The abdominal pain started one day prior to presentation and was described as generalized, constant, non-radiating, not aggravated or relieved by anything, and moderate in severity. The pain was associated with multiple episodes of bilious vomiting and reduced appetite. Additionally, the patient also had experienced constipation for three days prior to the presentation. On triage, he was found to have a national early warning score (NEWS) of 5 with a heart rate of 125 beats per minute, respiratory rate of 29 breaths per minute, blood pressure of 132/89 mmHg, temperature of 36.4 degrees Celsius, a saturation of 96% on room air. On general look, he was lying in bed with pain but alert and orientated to time, place, and person. Cardiovascular and respiratory system examinations were unremarkable. On abdominal examination, there was no visible distension of the abdomen, but generalized tenderness with guarding was noted on palpation. Percussion of the abdomen revealed a hyper-resonant abdomen, and bowel sounds were absent on auscultation. To complete the abdominal examination, a digital rectal examination was done with a chaperone present, which showed no stool in the rectum, no melaena, and no fresh bleed. His body mass index (BMI) was calculated to be 22.6. The patient’s past medical history included obstructive sleep apnoea (for which he used a continuous positive airway pressure mask) and osteoarthritis. He did not have any past surgical history of note. He is a retired gentleman living in his home with his wife and children. He often worked in the garden as a hobby. His alcohol intake was limited to occasional instances of consuming less than four units per week, and he had never smoked.

**Figure 1:**
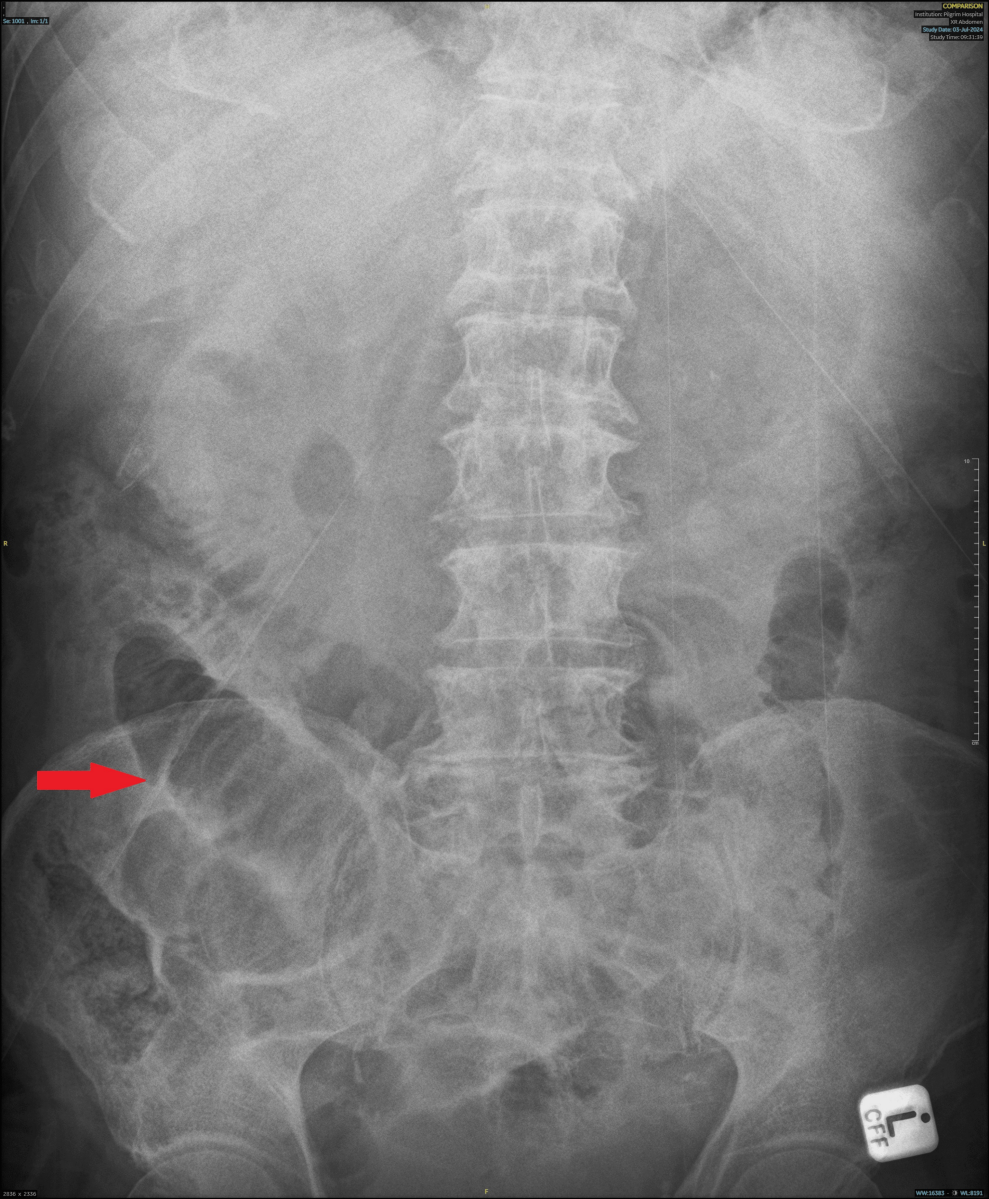
Abdominal X-ray. Dilated gas filled loops of small bowel measuring up to 4.2 cm are seen in the right flank.

**Figure 2:**
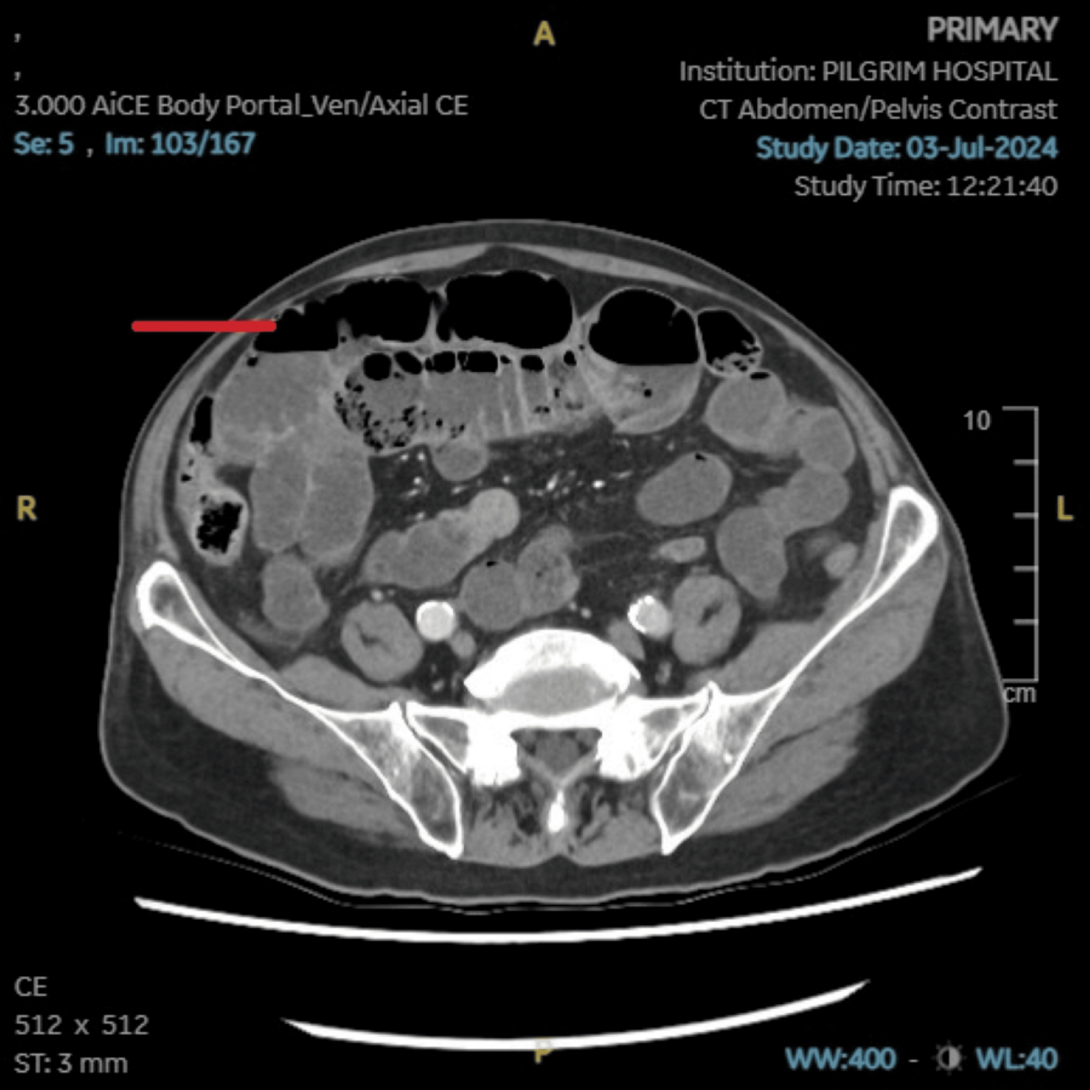
CT Abdomen pelvis with contrast. There is dilatation of the small bowel loops, with fluid distension of the stomach in the lower thoracic oesophagus. The distal small bowel loops are collapsed. The zone of transition appears to be in the mid abdomen (pointed red line).

**Figure 3:**
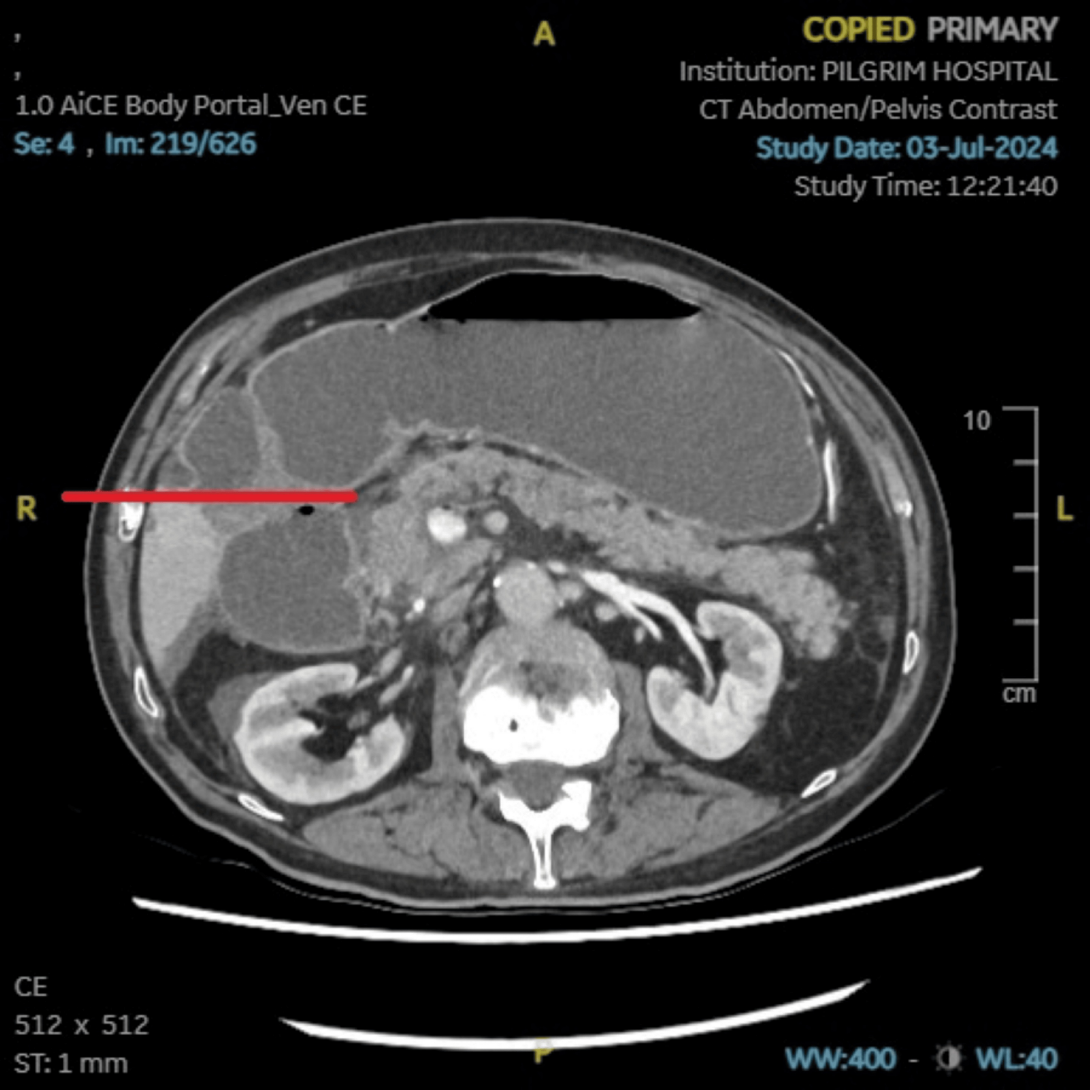
CT abdomen pelvis with contrast. Pancreas appears normal in size with subtle ill-defined hypodensity in the neck region associated with peripancreatic fluid extending to the superior recess of the lesser sac (pointed with red line).

Initial investigations included blood tests (Table 1), electrocardiogram (ECG) (Table 2), chest X-ray (Table 2), and abdominal X-ray (Figure 1 and Table 2). Based on the result of these investigations, the patient was commenced on pancreatitis and small bowel obstruction treatment protocol. Following the initial findings, a CT abdomen pelvis with contrast (Figure 2, 3, and Table 2) was requested. Subsequently, a CT angiogram (Table 2) was later ordered by the surgical team to identify the vessel implicated in bowel ischemia. Additionally, MRI small bowel (Table 2) was also requested to rule out the possibility of the neoplastic process.

**Table 1:** Blood investigations. Blood investigations that were taken at the time of admission: pCO_2_: partial pressure of carbon dioxide; pO_2_: partial pressure of oxygen; HCO_3_: bicarbonate ion.

Bloods	Results	Reference Values
Haemoglobin	168	135-160 (g/L)
White blood cell	21.2	4.0-11.0 *10^9^(/L)
Neutrophils	19.02	2.0-7.0 *10^9^(/L)
Platelet	410	150-400 *10^9^(/L)
C-reactive protein	290	<5 (mg/L)
Sodium	135	135-145 (mmol/L)
Potassium	3.6	3.5-5.5 (mmol/L)
Urea	21.6	2.0-7.0 (mmol/L)
Creatinine	110	55-120 (mmol/L)
Glomerular filtration rate	52	90-200 (mL/min)
Albumin	29	30-50 (g/L)
Amylase	1613	70-300 (u/L)
Lactate dehydrogenase	287	140-280 (u/L)
Adjusted calcium	2.54	2.1-2.6 (mmol/L)
pH	7.532	7.35-7.45
pCO_2_	4.23	4.5-6.0 (kPa)
pO_2_	6.43	10-14 (kPa)
HCO_2_	26.6	22-28 (mmol/L)
Lactate	3.5	0.5-2.2 (mmol/L)
Base excess	4.6	-2 to +2 (mmol/L)
Glucose	11.8	3.0-6.0 (mmol/L)

**Table 2:** Other investigations. Interpretation of the reports of other investigations.

Investigations	Report
ECG	Sinus rhythm with tachycardia
X-ray chest	No acute pathology was identified in the chest X-ray
X-ray abdomen	Dilated gas-filled loops of small bowel measuring up to 4.2 cm are seen in the right flank. The large bowel appears to have collapsed. Although some gas is seen in the rectum. Appearances are in keeping with small bowel obstruction.
CT abdomen pelvis with contrast	Subtle ill-defined hypo enhancement involving the neck of the pancreas with peripancreatic fluid extending to the superior recess of the lesser sac. Presence of air within the portal vein and its branches in the left lobe of the liver along with fluid-distended esophagus, stomach, and small bowel loops with a zone of transition in the mid-abdomen. The collapsed distal small bowel loops suggest small bowel obstruction. In view of the above finding, the possibility of acute pancreatitis and underlying ischaemic small bowel disease merits exclusion. Incidental findings of hypo-enhancing areas involving the bilateral adrenal gland and linear to wedge-shaped hypodensity involving the splenic parenchyma.
CT angiogram	Mid-small intestinal obstruction with an appearance suggesting multiple strictures, which in the presence of the multiple soft tissue masses would suggest a neoplastic process, and a small intestinal primary (one possibility is the anterior wall of the mid-third part of the duodenum) should first be excluded.
MRI small bowel	Changes in resolving pancreatitis with mild oedematous changes in the neck, such as effaced pancreatic duct in the neck region and mild prominence of the pancreatic duct in the body and tail. A small organized pocket of fluid collection in the mesenteric region, causing mild displacement of the adjacent jejunal loop with slight prominence of the loop of the jejunum proximal to it. No other significant abnormality involving the small bowel loops.

After an initial assessment of the patient, the patient was commenced on intravenous fluids in the form of normal saline, analgesia in the form of intravenous paracetamol, and intravenous ondansetron as an antiemetic. Based on the initial results, the patient was referred to the surgical team and was started on acute pancreatitis guidelines, which included keeping the patient nil per oral as part of bowel rest, giving intravenous fluids, pain management, and anti-emetics only. Furthermore, a nasogastric tube was later passed by the surgical team to decompress the intestines as part of a small bowel obstruction. The patient was discharged three weeks after the initial presentation following an improvement in his biomarkers and the reintroduction of oral intake.

## Discussion

This case highlights the complex relationship between acute pancreatitis, small bowel obstruction (SBO), splenic infarct, bowel ischaemia, and neoplasm as important factors to rule out. The following discussion will explore in great detail the links between these conditions. 

Bowel complications of acute pancreatitis, such as paralytic ileus, ischemic necrosis, perforation, and mechanical obstruction, are relatively infrequent [4,5]. Mechanical bowel obstruction as a result of acute pancreatitis has been described in the literature and is more likely to occur in the splenic flexure and transverse colon. This is believed to stem from either severe inflammation of the body and tail of the pancreas causing extrinsic compression, retroperitoneal extravasation of pancreatic enzymes causing pericolitis and/or pericolic fibrosis, thrombosis of mesenteric arteries (often associated with hypercoagulability during severe inflammatory states), infarction/ischemic necrosis of watershed areas secondary to systemic hypotension [6, 7-8]. Retroperitoneal inflammation may also lead to the involvement of other segments of the bowel, including the small intestine, as was the case with our patient. Small bowel obstruction has not been frequently described in the literature but most likely involves similar pathogenic mechanisms described above [9-14].

There has been evidence of acute pancreatitis causing SBO and vice versa. They suggest structural causes such as stenosis or extrinsic compression to be the main cause of obstruction, which may lead to SBO and acute pancreatitis [15,16]. SBO due to acute pancreatitis is generally managed with conservative management, which is the case in this patient. However, immediate surgery is required in cases of retroperitoneal collections or necrotizing pancreatitis [15]. Generally, the mortality rate for small bowel obstruction is 10%, but if there is bowel perforation or necrosis, the mortality rate increases significantly to 30% [17].

The location of the obstruction plays a factor in affecting its nearby structures. In this case, the more common location of bowel obstruction is in the splenic flexure and transverse colon [15]. This patient’s CT showed involvement of the spleen, whereby a splenic infarct is seen. Needless to say, there is a positive correlation between splenic infarct and mesenteric ischemia. Splenic infarct is a condition commonly seen in acute pancreatitis. Mortelé et al. studied CT scans in patients with acute pancreatitis, whereby they found 10% with splenic infarction [18]. 

CT severity index (CTSI) of pancreatitis can be used to link the severity of pancreatitis and the presence of splenic vein infarction/thrombosis/occlusion as more splenic vascular involvement leads to increased severity of acute pancreatitis [18]. The CTSI sums two scores, i.e., Balthazar score, which is based on grading of pancreatitis (A-E) and grading the extent of pancreatic necrosis on a CT scan. The Balthazar score was originally used alone, but the addition of a score for pancreatic necrosis improved its correlation with clinical severity scores. Together with the Ranson criteria, the severity and mortality of the patient can be calculated based on initial and 48-hour lab values (Table 3, 4).

**Table 3:** Ranson criteria. Ranson criteria for pancreatitis [19]. pO_2_: partial pressure of oxygen.

At admission	48 hours after admission
Age>55	Haematocrit fall>10%
White blood cells>16,000mm3	Blood urea nitrogen rise>5mg/dL
Glucose>200mg/dL	Calcium<8 mg/dL
Lactate dehydrogenase>350 IU/L	pO_2_<60 mmHg
Aspartate aminotransferase>250	Base excess>4
	Fluid sequestration>6L

**Table 4:** Ranson score and mortality Mortality according to Ranson score [19]. If score is less then 3, severe pancreatitis is unlikely.

Ranson score	Mortality
Score 0 to 2	2% mortality
Score 3 to 4	15 % mortality
Score 5 to 6	40% mortality
Score 7 to 8	100% mortality

At the time of admission, Ranson's score was calculated to be 4, indicative of acute pancreatitis, which could evolve into severe pancreatitis if not treated promptly. The CTSI sum score of our patient was 3 due to the peri-pancreatic fluid on CT finding and no evidence of necrosis. Together with the Ranson score, this suggested a diagnosis of mild acute pancreatitis with multiple complications and an estimated 15% mortality if not treated appropriately. These scores highlighted the high likelihood of the patient developing local complications such as pancreatic necrosis, pseudocyst, abscess, fistulae, thrombosis, or haemorrhage and systemic complications such as multiorgan failure, sepsis, acute kidney injury, and acute respiratory distress syndrome [1]. 

This study had limitations, including the unavailability of relevant blood investigations after 48 hours, which hindered the accurate calculation of the Ranson score.

## Conclusions

Patients coming in with acute abdominal pain can have a wide variety of symptoms and underlying diagnoses. In this case, the initial diagnosis of acute bowel obstruction was subsequently confirmed to be due to acute pancreatitis. It is essential in such cases to have a multi-disciplinary team approach utilized as early as possible. This involves collaboration among medical, surgical, gastroenterologists, radiologists, and intensivists to provide comprehensive management with the main to reduce patient mortality. Surgical management should be reserved for patients who exhibit complicated disease progression.

## References

[1] Johnson CD, Besselink MG, Carter R (2014). Acute pancreatitis. BMJ.

[2] Goodchild G, Chouhan M, Johnson GJ (2019). Practical guide to the management of acute pancreatitis. Frontline Gastroenterol.

[3] Gardner A, Gardner G, Feller E (2003). Severe colonic complications of pancreatic disease. J Clin Gastroenterol.

[4] Russell JC, Welch JP, Clark DG (1983). Colonic complications of acute pancreatitis and pancreatic abscess. Am J Surg.

[5] Lukash WM, Bisp RP (1967). Acute pancreatitis affecting the transverse colon. Report of a case. Am J Dig Dis.

[6] Aldridge MC, Francis ND, Glazer G, Dudley HA (1989). Colonic complications of severe acute pancreatitis. Br J Surg.

[7] Yoo SS, Choi SK, Lee DH (2008). [A case of colon obstruction developed as a complication of acute pancreatitis]. Korean J Gastroenterol.

[8] Miln DC, Barclay TH (1952). Acute colonic obstruction due to pancreatitis. Lancet.

[9] Pyun DK, Kim KJ, Ye BD (2009). [Two cases of colonic obstruction after acute pancreatitis]. Korean J Gastroenterol.

[10] Agrawal NM, Gyr N, McDowell W, Font RG (1974). Intestinal obstruction due to acute pancreatitis. Case report and review of literature. Am J Dig Dis.

[11] Brust R Jr, Chen KC (1962). Acute hemorrhagic pancreatitis complicated by duodenalobstruction. Report of a case. Am J Roentgenol Radium Ther Nucl Med.

[12] Sheikh H (1965). Duodenal ischaemia complicating acute pancreatitis. Br Med J.

[13] Street DF (1959). Duodenal obstruction associated with acute pancreatitis of traumatic origin. Br J Radiol.

[14] Moore TC (1956). Jejunal obstruction as a complication of acute hemorrhagic pancreatitis; report of a case. AMA Arch Surg.

[15] Sunkara T, Etienne D, Caughey ME, Gaduputi V (2017). Small bowel obstruction secondary to acute pancreatitis. Gastroenterology Res.

[16] Ooe Ooe, Y Y (2020). Severe acute pancreatitis caused by adhesive intestinal obstruction following fundoplication. J Ped Surg Case Rep.

[17] Mortelé KJ, Mergo PJ, Taylor HM, Ernst MD, Ros PR (2001). Splenic and perisplenic involvement in acute pancreatitis: determination of prevalence and morphologic helical CT features. J Comput Assist Tomogr.

[18] Xie CL, Zhang M, Chen Y (2018). Spleen and splenic vascular involvement in acute pancreatitis: an MRI study. Quant Imaging Med Surg.

[19] Basit H, Ruan GJ, Mukherjee S (2024). Ranson Criteria [Updated 2022 Sep 26]. In: StatPearls [Internet].

